# Loss of p21 does not protect against premature ovarian insufficiency caused by alkylating agents

**DOI:** 10.3389/fendo.2025.1616965

**Published:** 2025-07-16

**Authors:** Xiaohui Lu, Yongli Han, Jiaming Song, Qin Wan, Pengfei Liu, Li Chen, Yufeng Wang, Pingping Xue, Xiuliang Dai

**Affiliations:** ^1^ The Center for Reproductive Medicine, Changzhou Maternal and Child Health Care Hospital, Changzhou Medical Center, Nanjing Medical University, Changzhou, Jiangsu, China; ^2^ Department of Nursing, Changzhou Hygiene Vocational Technology College, Changzhou, Jiangsu, China; ^3^ The Department of Animal Center, Kebiao Medical Testing Center, Changzhou, Jiangsu, China

**Keywords:** p21, cellular senescence, premature ovarian insufficiency, alkylating agents, defective folliculogenesis, primordial follicle activation

## Abstract

**Background:**

Recent studies have focused on investigating the role of cellular senescence in ovarian aging. Targeting cellular senescence has been proposed as a potential strategy to improve ovarian aging. p16 and p21 are classical molecules involved in mediating cellular senescence. In our previous study, we demonstrated that ablation of p16 is dispensable for premature ovarian aging induced by alkylating agents. In the present study, we investigated whether p21 deficiency could mitigate ovarian aging caused by alkylating agents.

**Methods:**

Eight-week-old wild-type (WT, n=7) and p21 knockout (KO, n=7) female mice received a single injection of busulfan (BUL, 30 mg/kg) and cyclophosphamide (CTX, 120 mg/kg) to induce premature ovarian insufficiency (POI). Untreated WT (n=4) and p21 KO (n=4) mice served as controls. Ovaries were analyzed thirteen weeks after treatment. Ovarian reserve, folliculogenesis, cell proliferation, apoptosis and senescence, multinucleated giant cells (MGCs) and their characteristics, pro-inflammatory factors, fibrosis, ovarian stromal cell properties, and the expression of cell cycle inhibitors, including p16, p19, p27, and p53, were evaluated.

**Results:**

Female mice treated with alkylating agents exhibited typical features of POI, including a dramatic reduction in the number of primordial and growing follicles; defective folliculogenesis characterized by growth arrest in early-stage follicles, extensive atresia in mid-stage follicles, dysregulated FSH receptor (FSHr) expression in antral follicles, and abnormal over-activation of primordial follicles; the presence of hemosiderin-laden MGCs and fibrosis in the ovarian cortical region. p21 deficiency did not significantly mitigate these phenotypes. There were no significantly differences in the expression of pro-inflammatory factors, folliculogenesis-regulating factors, or steroidogenesis-related factors and cell cycle inhibitors between WT and p21 KO mice treated with alkylating agents. In addition, p21 deficiency did not prevent alkylating agent-induced cellular senescence.

**Conclusion:**

These results demonstrated that p21 is dispensable for POI caused by alkylating agents, suggesting that targeting p21 alone may not mitigate ovarian aging caused by alkylating agents.

## Introduction

The use of alkylating agents for cancer treatment is one of the primary causes of ovarian function decline and ovarian insufficiency (1). Alkylating agents-induced ovarian reserve decline in female mice, has been widely used as a mouse model of premature ovarian insufficiency in research (2–4). Based on our previous observations, we deduce that the ovarian phenotype of 5-month-old mice treated with busulfan (BUL) and cyclophosphamide (CTX) at 2 months of age resembles the aged ovaries of 17-month-old mice (5, 6). It has been reported that alkylating agents can directly induce cell death by crosslinking DNA strands, particularly in cells undergoing active DNA synthesis (7). Consequently, growing follicles with intensified DNA synthesis within granulosa cells are especially vulnerable to these drugs (8). More importantly, it has been reported that alkylating agents reduce the reserve of primordial follicles through either direct destruction or a “burnout” effect (8–10). In fact, a single dose of BUL+CTX is sufficient to induce the mouse model of premature ovarian insufficiency (11, 12). Considering the rapid metabolism of these drugs *in vivo*, their cytotoxic effects on growing follicles are relatively short-term. Therefore, it remains to uncover the mechanisms underlying the persistent damage to ovarian reserve following one injection of alkylating agents in mice.

The molecular mechanisms underlying ovarian aging have been intensively studied in recent years (13). Recent researches have focused on the role of cellular senescence and related pathways in driving ovarian aging (14). Several studies have demonstrated the accumulation of senescent cells in the ovary with aging, suggesting that these cells may contribute to ovarian aging (15, 16). It has been demonstrated that clearance of senescent cells can delay the process of ovarian aging caused by cisplatin (17). p16-dominated p16-pRB pathway and the p21-dominated p53-p21 pathway are two well-established cellular senescence pathways (18). It has been reported that the expression of p16 and p21 is significantly elevated in the ovaries of naturally aged or POI mice (19–21). However, in our previous study using alkylating agents-induced mouse model of ovarian aging, we found that deficiency in the p16 gene had no impact on premature ovarian aging caused by alkylating agents (5), indicating that activation of the p16 pathway in response to alkylating agents has little influence on ovarian aging. This has inspired us to consider whether it is the p21 signaling pathway, rather than the p16 pathway, that plays a central role in promoting ovarian aging in response to alkylating agents exposure.

In the present study, we utilized a p21 knockout mouse model to investigate whether inactivation of the p21 gene confers resistance to ovarian aging in female mice induced by alkylating agents. Measurements including ovarian reserve, folliculogenesis, cell proliferation, apoptosis and senecence, inflammation-related factors, multinucleated giant cells (MGCs) and related characteristics, fibrosis, properties of ovarian stromal cells and the expression of cell cycle dependent inhibitors, were examined.

## Materials and methods

### Mouse and treatment

The p21 heterozygous (p21^+/-^) mice were purchased from Cyagen Biosciences Company (C57BL/6J-Cdkn1a^em1Cya^, China). A p21 knockout mouse model was generated using a gRNA-guided gene knockout strategy. DNA sequencing confirmed a 1,921 bp deletion within exon 2 of the p21 gene (**Supplementary Figure 1A**). Genotyping was performed using two primer pairs: F1/R1 and F3/R1. The F1/R1 pair amplified a 609 bp fragment specific to the knockout (KO) allele, while the F3/R1 pair amplified a 644 bp fragment specific to the wild-type (WT) allele. The sequences of gRNA-A1, gRNA-A2, primer F1, primer R1, and primer F3, and DNA sequencing primer are listed in **Supplementary Table 1**. The genotyping results are shown in **Supplementary Figure 1B**. All mice were maintained in an SPF animal laboratory under standard conditions, with unrestricted access to food and water, and a 12-hour light/dark cycle. Male and female p21^+/-^ mice were bred to generate p21 homozygous (p21^-/-^) mice and their wild-type (WT) littermate controls. Eight-week-old female WT and p21^-/-^ mice were treated with BUL (30 mg/kg) and CTX (120 mg/kg) to establish an ovarian insufficiency model, as previously described. After thirteen weeks, the animals were euthanized via cervical dislocation, and ovaries, liver, lungs, and kidneys were harvested.

### Ovarian follicle counts

Ovaries were serially sectioned at 5 µm thickness. Beginning with the first section, every eighth section was collected for follicle counting until the last section of the ovary. The remaining sections were collected for histological or immunohistological analysis. Follicle classification followed established criteria: Primordial follicle: The oocyte is surrounded by a single layer of squamous granulosa cells. Primary follicle: The oocyte is surrounded by cuboidal granulosa cells. Secondary follicle: The oocyte is surrounded by two or more layers of granulosa cells. Antral follicle: An antral cavity is present. To avoid duplicate counting, only follicles with visible oocyte nuclei were included. The total number of follicles per ovary was calculated by multiplying the counted follicles by 5, as described in a previous study (22).

### Immunofluorescence and immunohistochemistry

Following deparaffinization and rehydration, antigen retrieval was performed in a pressure cooker for 10 minutes. For immunofluorescence, sections were incubated with 10% donkey serum to block nonspecific binding. The sections were incubated with primary antibodies, including anti-PCNA (13110T, Cell signaling technology), anti-F4/80 (28463-1-AP, Proteintech), anti-α-SMA (14395-1-AP, Proteintech), anti-FSHr (22665-1-AP, Proteintech), anti-Laminin B1 (17416T, Cell signaling technology), anti-Foxo3a (12829T, Cell signaling technology) and anti-Cyp11a1 (ab272494, Abcam), overnight at 4°C. Then, the sections were washed with PBS and incubated with Cy3-conjugated goat anti-rabbit secondary antibody (A0516, Beyotime Biotechnology, China) at room temperature (RT) for 1 hour. Finally, sections were counterstained with DAPI and mounted for imaging. For Immunohistochemistry: the sections were incubated after antigen retrieval, with 3% H_2_O_2_ for 30 minutes at RT to inactivate endogenous catalase. The sections were then incubated with anti-Caspase 3 (ab184787, Abcam), anti-p21 (ab18824, Abcam) and HRP-conjugated goat anti-rabbit secondary antibody (A0208, Beyotime Biotechnology, China). DAB substrate was used for visualization, and sections were counterstained with hematoxylin. After dehydration and clearing, slides were mounted for imaging.

### TUNEL assay

TUNEL assay was performed using the TUNEL BrightGreen Apoptosis Detection Kit (A112-01, Vazyme, China). After deparaffinization and rehydration, ovarian sections were incubated with Proteinase K (40 μg/mL) for 30 minutes at RT. The sections were then treated with equilibration buffer at RT for 20 minutes, followed by incubation with TdT reaction solution at 37 °C for 1 hour. Finally, the sections were stained with DAPI and mounted for imaging. DNase I-treated sections were used as positive control.

### Detection of autofluorescence in MGCs

The sections stained with H&E for follicle counting were directly examined under a fluorescence microscope to visualize MGC autofluorescence.

### Colocalization of Prussian blue staining and autofluorescence

Following deparaffinization and rehydration, sections were first examined under a fluorescence microscope and recorded. The same sections were then subjected to Prussian blue staining using a commercial kit (BP-DL161, SenBeiJia Biological Technology, China). Briefly, the sections were rinsed in distilled water for 1 minute. The sections were incubated with Perls stain solution for 30 minutes. The sections were rinsed in distilled water for 5 minutes, followed by neutral red solution for 5 minutes. After dehydration and clearing, slides were mounted for imaging.

### Picrosirius red staining

After deparaffinization and rehydration, sections were stained with PSR solution (R20385, Yuanye Biotechnology Company, China) for 6 hours at room temperature. Following dehydration and clearing, slides were mounted for imaging. The images of PSR staining were processed using ImageJ (Fiji version) to quantify the selected PSR-positive area and the selected area of the ovarian sections. The original images were used as references to set the threshold for distinguishing positive staining from background. For analysis of PSR-positive fibers in the entire ovarian section, both the total area of PSR-positive fibers and the total area of the ovarian section were measured. For analysis in the cortical region, both the PSR-positive area and the total area of the cortical region were measured. The percentage of PSR-positive staining was determined by dividing the PSR-positive area by the total selected area.

### Real-time qPCR

Total RNA from ovary, liver, lung, and kidney tissues was extracted using Freezol buffer (R711-01, Vazyme, China). A reverse transcription kit (R123-01, Vazyme, China) was used to synthesize cDNA from 500 ng of RNA per sample. Real-time qPCR was performed using a SYBR Green kit (Q331-02, Vazyme, China), with Gapdh as the reference gene. The 2^−ΔΔCT^ method was applied to calculate relative mRNA expression levels. Primer sequences for each gene are listed in **Supplementary Table 2**.

### Statistics

Data are presented as mean ± SD. Comparison among four groups: One-way ANOVA followed by Tukey’s multiple comparisons test was performed. Comparison between two groups: An unpaired Student’s t-test was used. Statistical significance was set at p < 0.05. All analyses and statistical graphing were conducted using Prism software (GraphPad).

## Results

### p21 deficiency did not ameliorate alkylating agents-induced ovarian reserve decline

**Figure 1:**
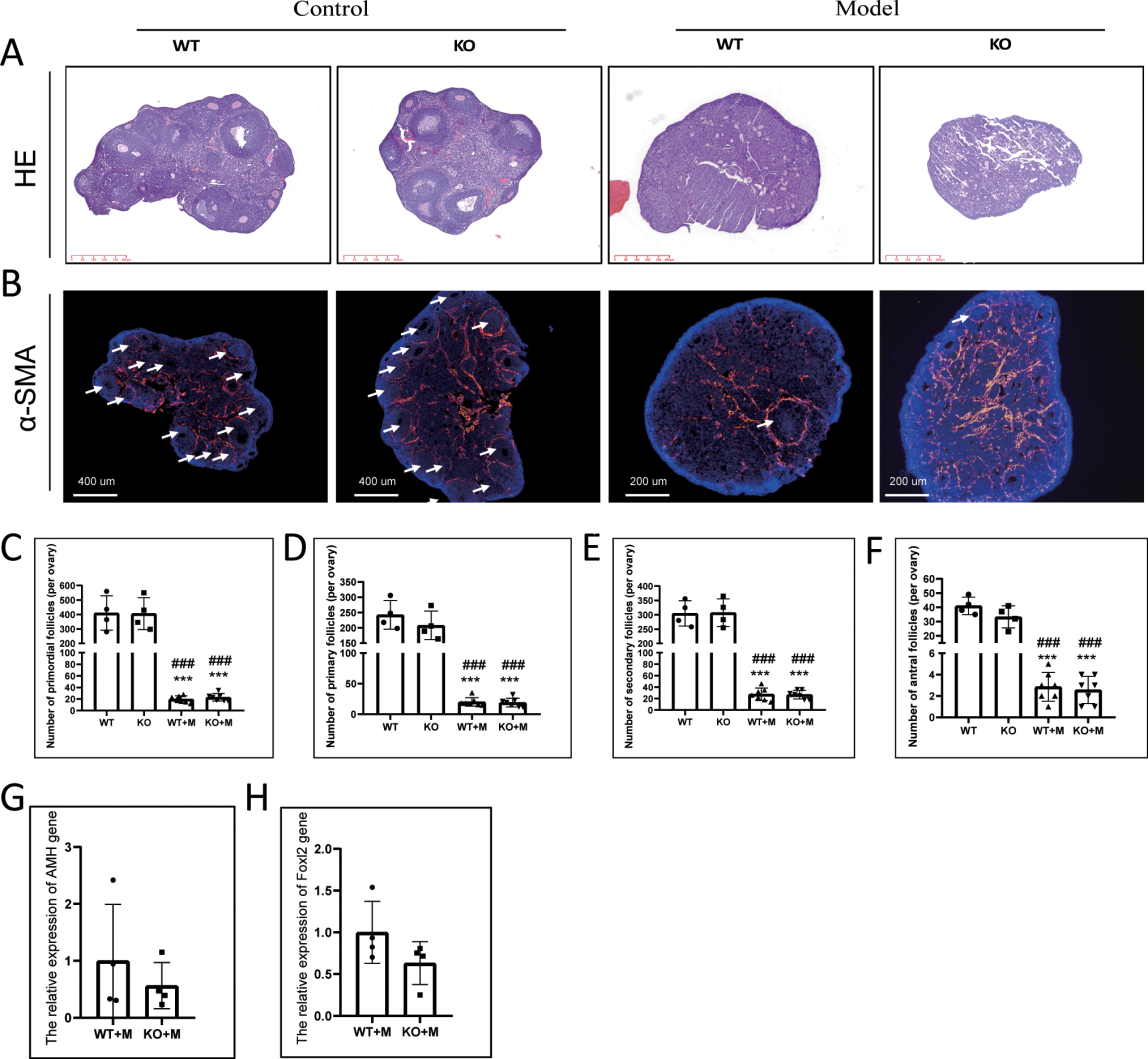
p21 deficiency did not prevent the decline in ovarian reserve caused by BUL+CTX treatment. **(A)** H&E staining of ovarian sections. N=3 for each group. **(B)** Immunofluorescent staining using an anti-αSMA antibody. White arrows indicate ovarian follicles surrounded by αSMA-positive staining. N=3 for each group. Quantification of **(C)** primordial follicles, **(D)** primary follicles, **(E)** secondary follicles, and **(F)** antral follicles per ovary. N=4 for untreated WT and KO mice, N=7 for WT and KO mice treated with BUL+CTX. Statistical analysis was performed using one-way ANOVA followed by Tukey’s multiple comparisons test. mRNA expression levels of **(G)** *Amh* and **(H)** *Foxl2* genes in the ovaries of WT and KO mice treated with BUL+CTX. N=4 for each group. Statistical analysis was performed using t-student test. Compared to untreated WT, ***p < 0.001; compared to untreated KO, ^###^p < 0.001. Model/M: mice treated with BUL+CTX; WT: wild-type; KO: p21 knockout.

Thirteen weeks after WT and p21 KO female mice were treated with BUL+CTX, the mice were sacrificed. No p21 mRNA or positive cells were detected in the ovaries of p21 KO mice, confirming complete depletion of the *p21* gene (**Supplementary Figures 2A, B**). Ovarian size and weight were significantly reduced in both WT and p21 KO mice treated with BUL+CTX compared to untreated controls (**Supplementary Figures 2C, D**). However, no significant difference in ovarian weight was observed between WT and p21 KO mice, regardless of treatment (**Supplementary Figure 2D**). Histological analysis via H&E staining revealed abundant follicles in untreated WT and p21 KO ovaries, while scarce follicles were observed in BUL+CTX-treated ovaries from both genotypes (**Figure 1A**). Alpha-smooth muscle actin (α-SMA), a well-established marker of smooth muscle cells and myofibroblasts, is highly expressed in ovary (23). Immunostaining for α-SMA, which can label follicles and corpus luteum (24), corroborated these findings (**Figure 1B**). Follicle counts showed a significant reduction in primordial, primary, secondary, and antral follicles in ovaries from both WT and p21 KO mice treated with BUL+CTX, compared to untreated controls (**Figures 1C–F**). No significant difference in follicle counts at any stage was observed between WT and p21 KO mice treated with BUL+CTX (**Figures 1C–F**). Furthermore, mRNA expression levels of *Amh* (a marker of antral follicles) and *Foxl2* (a marker of granulosa cells) were similar between WT and p21 KO mice treated with BUL+CTX (**Figures 1G, H**). Collectively, these results suggested that p21 deficiency did not protect against the ovarian reserve decline induced by alkylating agents.

### p21 deficiency did not prevent abnormal primordial follicle overactivation or follicular atresia induced by alkylating agents

**Figure 2:**
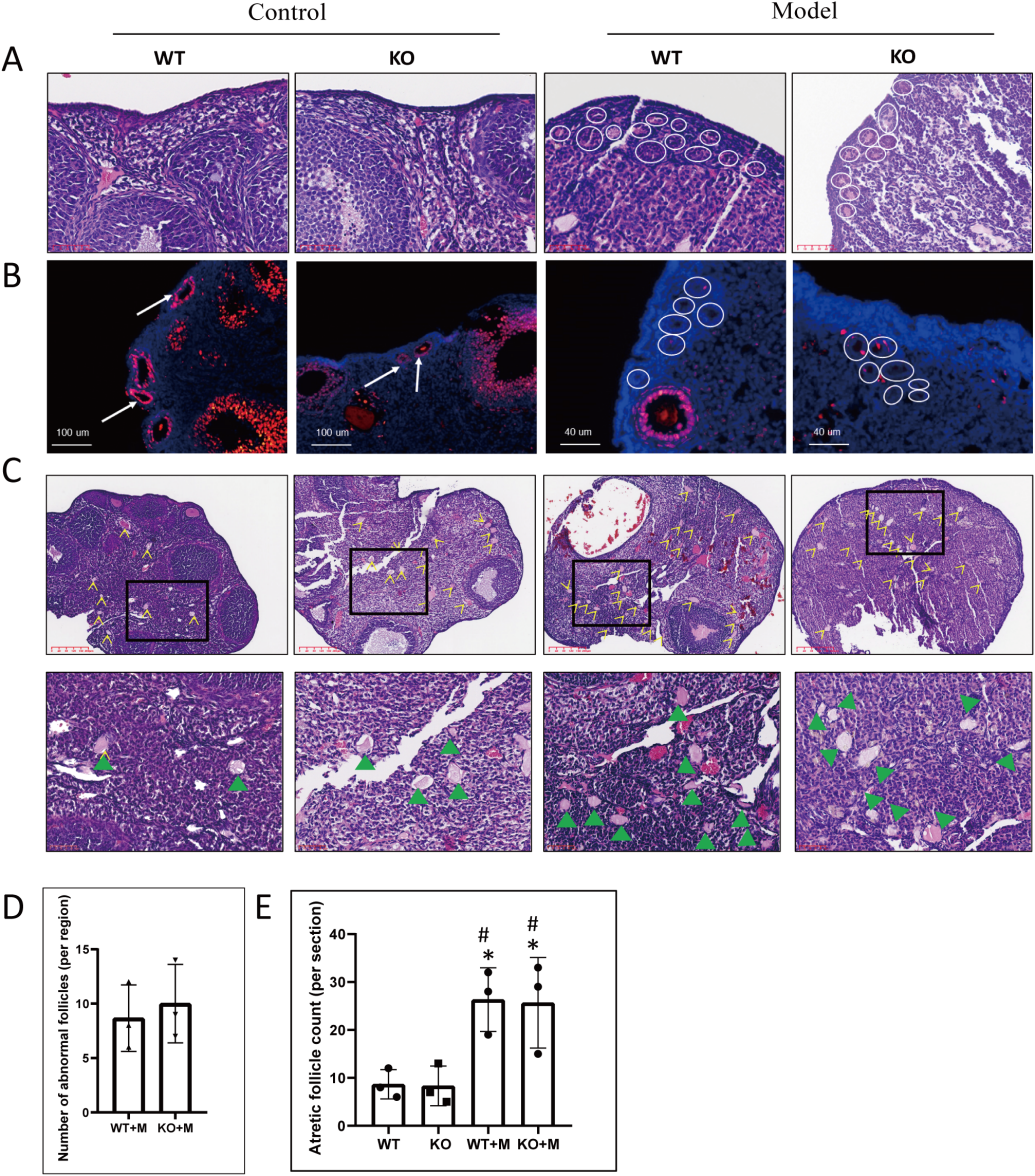
p21 deficiency did not prevent primordial follicle over-activation or follicular atresia caused by BUL+CTX treatment. **(A)** H&E staining of ovarian sections. White circles indicate primary and transitional ovarian follicles without visible nuclei in BUL+CTX-treated WT or p21 KO mice. N=3 for each group. **(B)** Immunofluorescent staining using an anti-PCNA antibody. White arrows indicate PCNA-positive primary follicles in untreated WT or p21 KO mice, while white circles highlight primary and transitional ovarian follicles without visible nuclei in BUL+CTX-treated WT or p21 KO mice. N=3 for each group. **(C)** H&E staining showing atretic follicles, with yellow arrow indicating atretic follicles. The images below are magnified from the boxed area, and the green arrowheads indicate atretic follicle. N=3 for each group. **(D)** Graph depicting the number of abnormal follicles per centralized sub-area as shown in **(A)**. Statistical analysis was performed using t-student test. **(E)** Graph depicting the number of atretic follicles per ovarian section. Statistical analysis was performed using one-way ANOVA followed by Tukey’s multiple comparisons test. Compared to untreated WT, *p < 0.05; compared to untreated KO, ^#^p < 0.05. Model/M, mice treated with BUL+CTX; WT, wild-type; KO, p21 knockout.

Our previous findings have demonstrated that treatment with BUL+CTX induced the accumulation of multiple primary or transitional follicles without visible nuclei in the ovarian cortex (5), reflecting a “burnout” effect of primordial follicles. Similarly, in this study, primary or transitional follicles lacking visible nuclei were observed in the ovarian cortical regions of both WT and p21 KO mice treated with BUL+CTX, while these follicles were absent in untreated WT or p21 KO mice (**Figure 2A**). There was no significant difference in the number of these abnormal follicles between WT and p21 KO mice treated with BUL+CTX (**Figure 2D**). Further analysis showed that these follicles were negative for PCNA (**Figure 2B**). These results indicated that p21 deficiency did not prevent the overactivation of primordial follicles and subsequently growth arrest of early follicles in ovaries of female mice treated with BUL+CTX. Consistent with the overactivation of primordial follicles, Foxo3a-positive primordial follicles were readily observed in the ovaries of untreated WT and KO female mice, whereas no Foxo3a-positive follicles were detected in the ovaries of WT and p21 KO female mice treated with BUL+CTX (**Supplementary Figure 3**). Additionally, although atretic follicles were present in untreated WT and p21 KO mice, their numbers significantly increased in BUL+CTX-treated WT or p21 KO mice (**Figure 2C**). Specifically, these atretic follicles were located in the deep cortical region or medullary region, and characterized by degenerative oocytes without distinguishable follicle structure (**Figure 2C**), indicating mid-stage follicular developmental arrest. However, no significant difference in atretic follicle numbers was observed between WT and p21 KO mice, either with or without BUL+CTX treatment (**Figure 2E**). These results indicated that p21 deficiency did not mitigate primordial follicle overactivation and follicular atresia caused by alkylating agents.

### p21 deficiency did not rescue impaired folliculogenesis induced by alkylating agents

**Figure 3:**
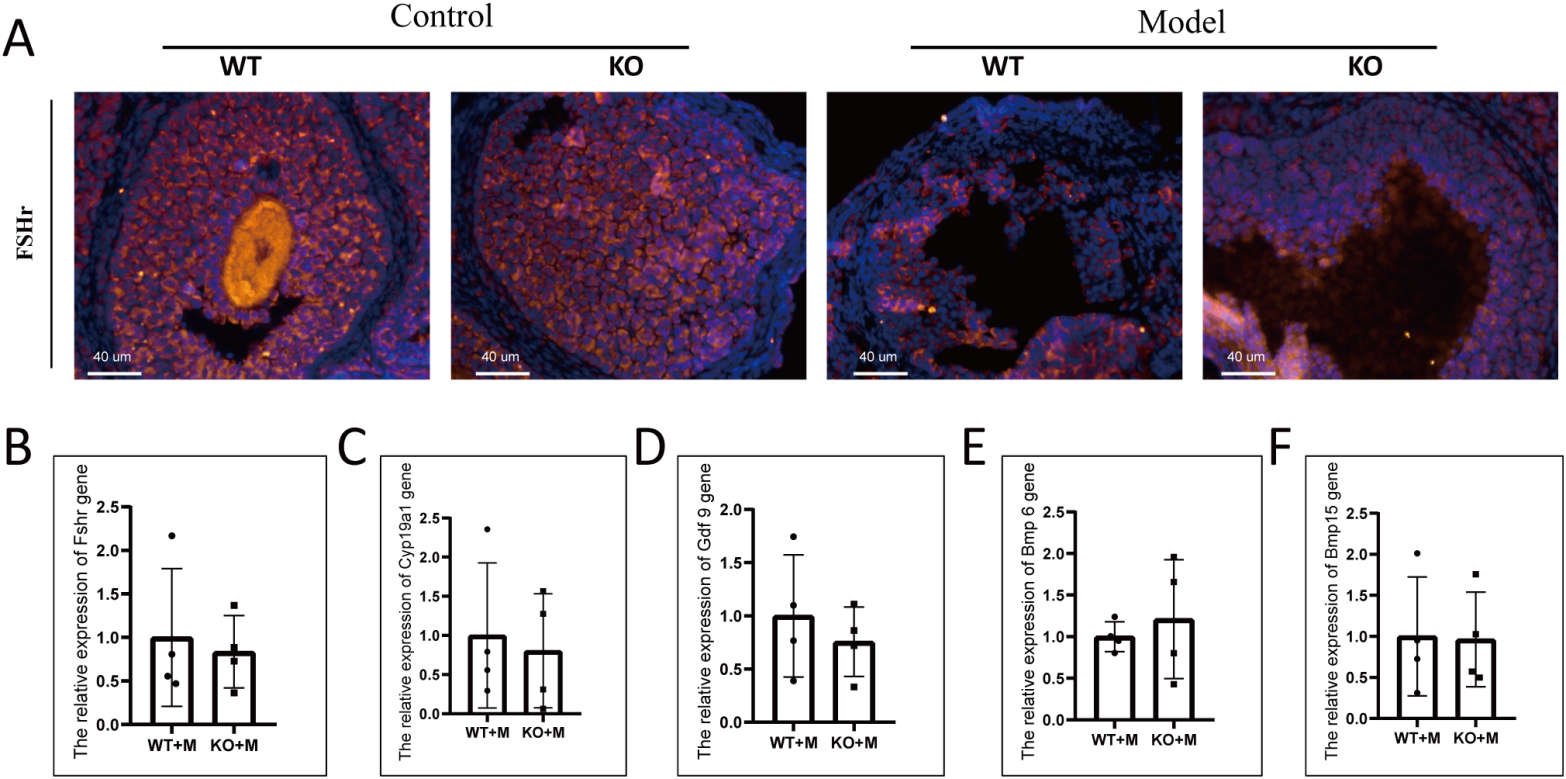
p21 deficiency did not prevent abnormal folliculogenesis caused by BUL+CTX treatment. **(A)** Immunofluorescent staining using an anti-FSHr antibody. N=3 for each group. mRNA expression levels of **(B)** *Fshr*, **(C)** *Cyp19a1*, **(D)** *Gdf9*, **(E)** *Bmp6*, and **(F)** *Bmp15* genes in the ovaries. N=4 for each group. Statistical analysis was performed using t-student test. Model/M: mice treated with BUL+CTX; WT: wild-type; KO: p21 knockout.

The number of follicles with PCNA-positive granulosa cells was significantly reduced in both WT and p21 KO mice treated with BUL+CTX, compared to untreated controls (**Supplementary Figure 4A**). No significant difference was observed between WT and p21 KO mice in the number of PCNA-positive follicles, regardless of treatment with or without BUL+CTX (**Supplementary Figure 4E**). The number of TUNEL- or Caspase-3–positive cells was comparable among the four groups (**Supplementary Figures 4B–D, F, G**). We then analyzed the expression of FSHr in morphologically intact follicles across groups. In untreated WT and p21 KO mice, FSHr-positive signaling was uniformly distributed throughout the granulosa cell layer of antral follicles. (**Figure 3A**). By contrast, FSHr staining exhibited an irregular distribution, with distinct bright and dark regions within antral follicles of both WT and p21 KO mice treated with BUL+CTX (**Figure 3A**). Further analysis of mRNA levels for *Fshr*, *Cyp19a1*, *Gdf 9*, *Bmp 6*, and *Bmp15* in ovaries, which are crucial for normal folliculogenesis, revealed no significant differences between WT and p21 KO mice treated with BUL+CTX (**Figures 3B–F**). Collectively, these results indicated that p21 deficiency did not mitigate the impaired folliculogenesis caused by alkylating agents.

### p21 deficiency did not prevent multinucleated giant cell formation in ovaries treated with alkylating agents

**Figure 4:**
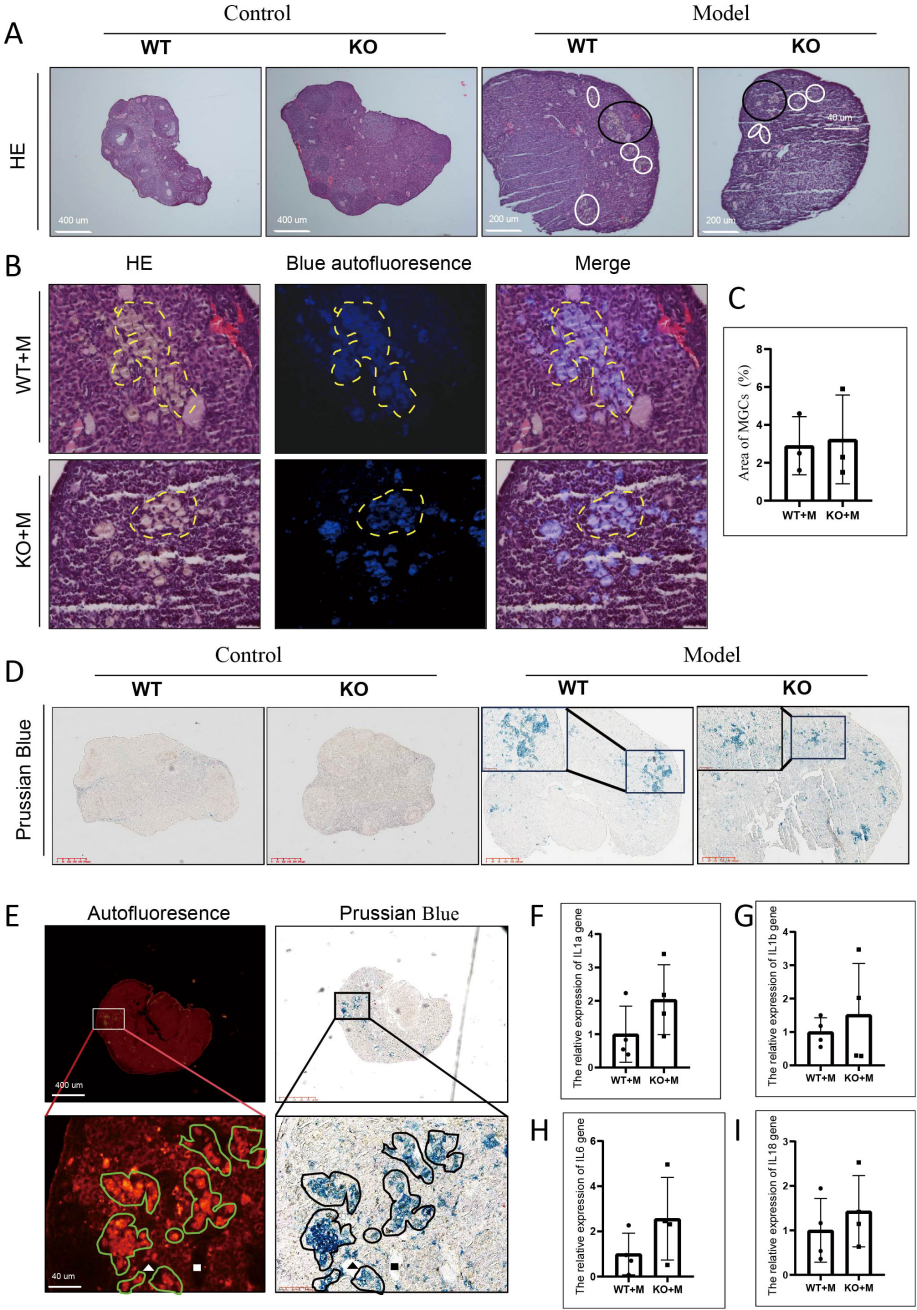
p21 deficiency did not prevent the occurrence of MGCs caused by BUL+CTX treatment. **(A)** H&E staining showing MGCs. White and black circles indicate MGCs. N=3 for each group. **(B)** The images are magnified from the black circle area in **(A)**, and shows a combined image of H&E staining with blue autofluorescence. The yellow dashed line outlines the MGCs. **(C)** Graph depicting the area occupied by MGCs per section. N=3 for each group. Statistical analysis was performed using t-student test. **(D)** Prussian Blue staining of ovarian sections, with the inset showing a high-magnification image of positive staining. N=3 for each group. **(E)** The left panel shows red autofluorescence, while the right panel shows Prussian Blue staining of the same section and region. Lower images are magnified from boxed region in upper images. Green irregular circles indicate MGCs, and black irregular circles indicate Prussian Blue-positive staining. The white and black triangles and square rectangles indicate the same position on the same sections. N=3 for WT or p21 KO mice treated with BUL+CTX. mRNA expression levels of **(F)** *Il1a*, **(G)** *Il1b*, **(H)** *Il6*, and **(I)** *Il18 genes* in the ovaries. N=4 for each group. Statistical analysis was performed using t-student test. Model/M: mice treated with BUL+CTX; WT: wild-type; KO: p21 knockout.

The formation of MGCs, a hallmark of ovarian aging (25), was observed in the ovaries of both WT and p21 KO mice treated with BUL+CTX, but not in untreated controls (**Figure 4A**). We previously showed that MGCs exhibited autofluorescence in naturally aged ovaries (6). We showed that the MGCs observed in ovaries of female mice treated with BUL+CTX also exhibited autofluorescence (**Figure 4B**). Immunofluorescence revealed that the MGCs were F4/80-positive and showed signs of proliferation (**Supplementary Figures 5A, B**). The percentage of MGC-occupied area in the ovarian sections was similar between WT and KO mice treated with BUL+CTX (**Figure 4C**). Prussian blue staining is used primarily to detect iron deposits, such as hemosiderin in tissues. Positive staining of Prussian blue was observed in the ovaries of both WT and p21 KO mice treated with BUL+CTX, but not in untreated controls (**Figure 4D**). Further analysis showed the colocalization of Prussian blue staining with MGCs (**Figure 4E**). It has been proposed that chronic inflammation is considered a hallmark of ovarian aging (26, 27). Our results showed that mRNA levels of pro-inflammatory factors (*Il1a*, and *Il1b*, *Il6* and *Il18*) in the ovaries were comparable between WT and p21 KO mice treated with BUL+CTX (**Figures 4F–I**). These findings indicated that p21 deficiency did not prevent the formation of MGCs and had no impact on inflammatory status in ovaries treated with alkylating agents.

### p21 deficiency did not affect ovarian cortical fibrosis in ovaries treated with alkylating agents

**Figure 5:**
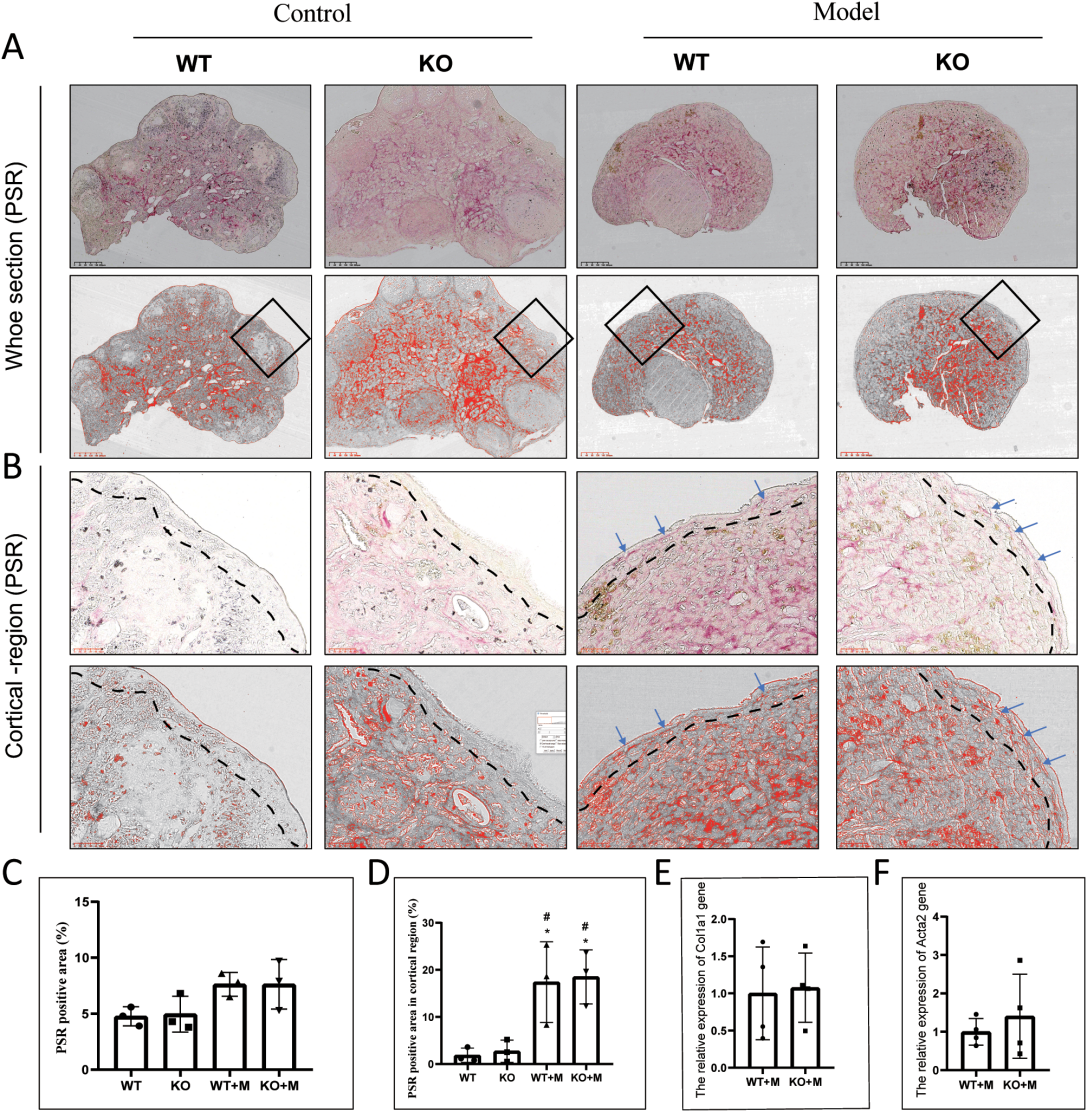
p21 deficiency did not affect ovarian fibrosis caused by BUL+CTX treatment. **(A)** PSR staining of the whole ovarian tissue. The upper panel shows original PSR staining, while the lower panel shows threshold images (for the purpose of presenting positive staining) processed by Image J. **(B)** The images are magnified from the black rectangle area in **(A)**.The upper panel shows original PRS staining, while the lower panel shows threshold images processed by Image J. Blue arrows indicate PSR-positive staining. N=3 for each group. Graph depicting **(C)** the PRS-positive area and **(D)** the PSR-positive area in cortical region. mRNA expression levels of **(E)** *Col1a1*, and **(F)** *Acta2*. N=4 for each group. Statistical analysis was performed using t-student test. Model/M, mice treated with BUL+CTX; WT, wild-type; KO, p21 knockout.

Ovarian fibrosis is considered as a marker of ovarian aging (28). Our results demonstrated that although ovarian follicles were scarce in both WT and p21 KO mice treated with BUL+CTX, the degree of fibrosis was similar across all groups, including untreated WT and p21 KO mice and those treated with BUL+CTX (**Figures 5A, C**). However, PSR-positive staining in the cortical region of the ovaries was observed in both WT and p21 KO mice treated with BUL+CTX, but not in untreated WT or p21 KO mice (**Figure 5B**). No significant difference in the PSR-positive area in the cortical region was observed between WT and p21 KO mice treated with BUL+CTX (**Figures 5B, D**). Furthermore, the expression levels of *Col1a1* and *Acta2* mRNA in the ovaries were similar between WT and p21 KO mice treated with BUL+CTX (**Figures 5E, F**). Previously, we observed high expression levels of steroidogenic factors such as CYP11A1, SF1, and STAR in ovarian stromal cells in naturally aged female mice (6). Here we also observed widespread expression of CYP11A1 in ovarian stromal cells, with comparable levels across all groups (**Supplementary Figure 6A, B**). Further analysis confirmed that the mRNA expression levels of *Cyp11a1*, *Sf1*, *Star*, and *Hsd3b1* in the ovaries did not differ significantly between WT and p21 KO mice treated with BUL+CTX (**Supplementary Figures 6C–F**). These findings indicated that treatment with BUL+CTX did not significantly affect whole-ovary fibrosis or the expression of ovarian stromal cell markers, but it did induce fibrosis specifically in the ovarian cortical region. However, the fibrosis in ovarian cortical region caused by BUL+CTX treatment was independent of p21.

### p21 deficiency did not impact ovarian cellular senescence or alter the expression levels of other cyclin-dependent kinase inhibitors in ovaries treated with alkylating agents

**Figure 6:**
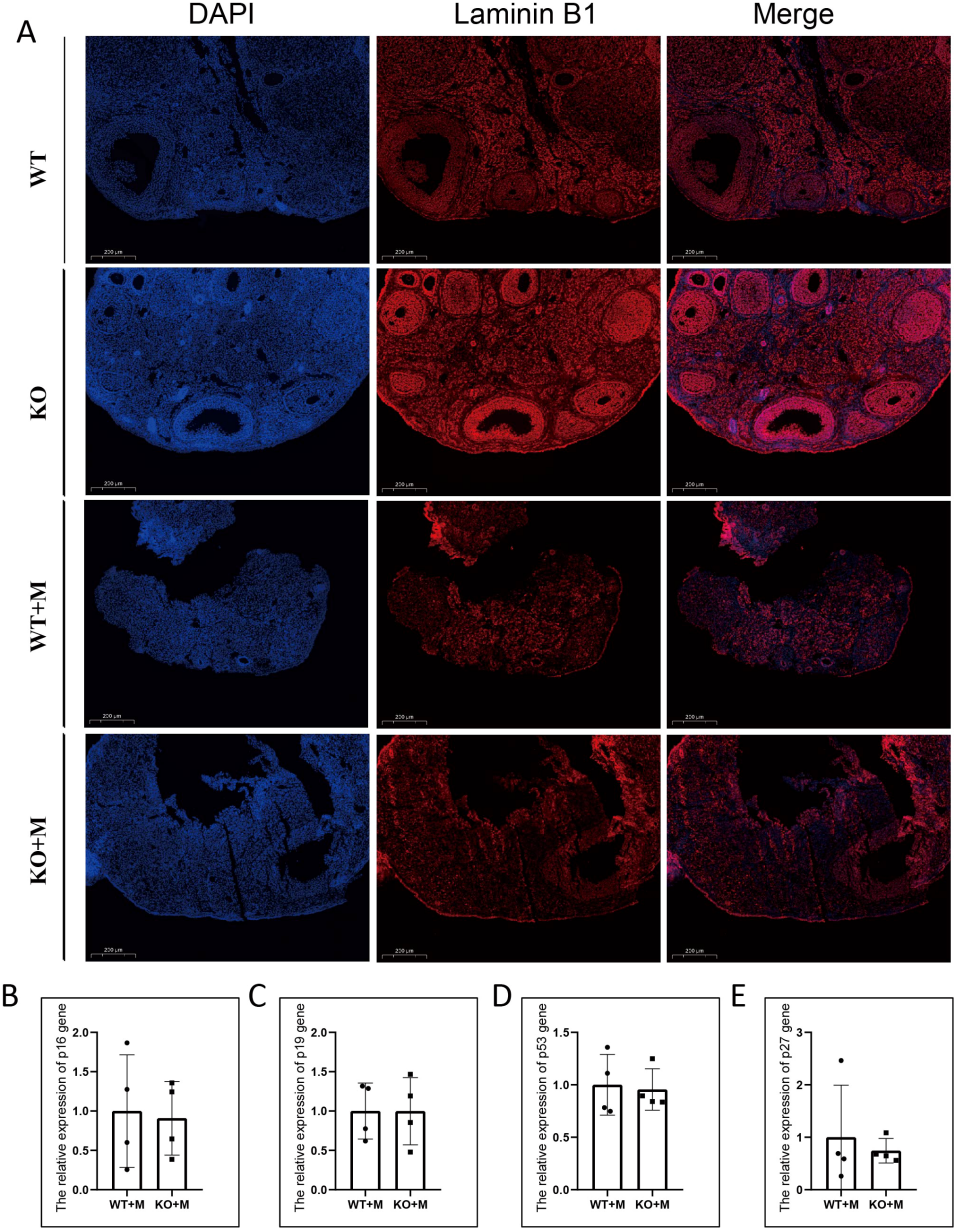
p21 deficiency did not impact ovarian cellular senescence or alter the expression levels of other CDKIs in ovaries treated with alkylating agents. **(A)**  Immunofluorescent staining using an anti-Laminin B1 antibody. N=4 for each group. **(B–E)** mRNA expression levels of **(B)** *p16*, **(C)** *p19*, **(D)** *p53*, and **(E)** *p27* in the ovaries. N=4 for each group. Statistical analysis was performed using t-student test. M, mice treated with BUL+CTX; WT, wild-type; KO, p21 knockout.

To determine whether p21 deficiency alleviated ovarian cell senescence induced by BUL+CTX treatment, we performed immunofluorescence staining for Lamin B1, a marker of cellular senescence, on ovarian sections from different groups. Compared to untreated WT and KO mice, Lamin B1 intensity was significantly reduced in the ovarian sections of both WT and KO mice treated with BUL+CTX (**Figure 6A**). However, no significant difference in Lamin B1 intensity was observed between WT and KO mice following BUL+CTX treatment (**Figure 6A**). It is possible that other cell cycle inhibitors might be up-regulated to functionally compensate for p21 loss, thereby maintaining cell senescence. To explore this, we analyzed the expression of other CDKIs, including *p16*, *p19*, *p27*, and *p53*, in WT and p21 KO ovaries treated with BUL+CTX. Our results demonstrated no significant differences in the expression of these genes between WT and p21 KO mice treated with BUL+CTX (**Figures 6B–E**). The p21 knockout mouse model used in our study is a total knockout model. To further verify the results from ovary, we examined the expression of these genes in other organs, including the liver, lung, and kidney, in WT and p21 KO female mice treated with BUL+CTX. Consistently, there were no significant differences in the expression of these genes in these organs between WT and p21 KO mice treated with BUL+CTX (**Supplementary Figures 7A–L**). Collectively, these results indicate that p21 deficiency does not affect the expression of other CDKIs following treatment with alkylating agents.

## Discussion

In this study, we demonstrate for the first time that the ablation of the *p21* gene does not attenuate alkylating agent-induced ovarian aging. Furthermore, no compensatory upregulation of other CDK inhibitors, such as *p16* gene, was observed in the ovary following alkylating agent exposure. These findings suggest that p21 is not essential for ovarian aging induced by alkylating agents. Consequently, targeting p21 alone may not be a feasible strategy for preserving female fertility in cancer patients.

Primordial follicles serve as the foundation for folliculogenesis (29). Previous studies have demonstrated a critical role of p27 in maintaining the primordial follicle pool, as p27 deficiency leads to premature ovarian insufficiency in mice due to accelerated primordial follicle activation (30, 31). In this study, we found no significant differences in the number of primordial or growing follicles between WT and p21 KO mice. This finding aligns with previous studies reporting normal fertility in female p21 KO mice (32). Therefore, unlike p27, p21 is not essential for preventing primordial follicle over-activation.

The over-activation of primordial follicles has been proposed as a contributing factor to premature ovarian insufficiency induced by chemical agents (8, 9). Consistently, our study revealed an accumulation of abnormal early follicles, including primary and transition follicles, in the ovarian cortex of female mice treated with BUL+CTX. These abnormal follicles exhibited oocytes lacking viable nuclei and granulosa cells arrested in the cell cycle, suggesting primordial follicle over-activation and subsequent developmental arrest of early follicles. Additionally, we observed an increase in atretic follicles within the deep cortical and the ovarian medulla of BUL+CTX-treated mice, indicating impaired follicular growth at the middle stages of development. Based on these findings, we propose that defects in folliculogenesis, characterized by impaired early and mid-stage follicular growth, lead to a reduced number of antral follicles. This, in turn, triggers excessive primordial follicle activation, establishing a vicious cycle in which defective folliculogenesis drives further primordial follicle activation, ultimately contributing to BUL+CTX-induced premature ovarian insufficiency. However, p21 deficiency had no impact on this pathological cycle, indicating that p21 is not required for early and mid-stage follicular developmental arrest induced by BUL+CTX.

Clinical observations from *in vitro* fertilization have shown variability in the fertilization potential of mature oocytes derived from ovulated follicles (33). It is well established that the normal proliferation of ovarian granulosa cells is a prerequisite for the development of mature oocytes (34, 35). Previous studies have indicated the reduced proliferation of ovarian granulosa may contribute to POI (36–39). However, our study reveals that despite the scarcity of antral follicles in BUL+CTX-treated ovaries, these follicles exhibited normal granulosa cell proliferation and an absence of apoptosis, regardless of genotype. This finding is consistent with our previous reports (5, 12). Interestingly, rather than alterations in cell proliferation, we observed a reduction in FSHr expression and an abnormal localization of FSHr in antral follicles from BUL+CTX-treated ovaries. Given that antral follicle development is dependent on FSH-FSHr signaling (40), these findings suggest a compromised quality of antral follicles in BUL+CTX-treated ovaries. Furthermore, this implies that FSHr expression, rather than granulosa cell proliferation, may serve as a more accurate indicator of follicular quality. However, p21 deficiency did not affect the aberrant FSHr expression in antral follicles from BUL+CTX-treated mice. Therefore, p21 is not required for the dysregulated FSHr expression in antral follicles of BUL+CTX-treated mice.

The presence of MGCs in the ovary is considered as a hallmark of ovarian aging (25). In this study, we observed MGCs in the ovaries of mice treated with BUL+CTX. In addition to previously reported characteristics- such as autofluorescence, proliferative capacity, and a macrophage-like phenotype (F4/80 expression) (28)-we report for the first time that these MGCs are Prussian blue-positive, demonstrating the content of hemosiderin. The hemosiderin-laden macrophages have been documented in diffuse alveolar hemorrhage, where they play a role in digesting extravasated red blood cells (41). In addition, hematomas are frequent observed in aged ovaries (6). Thus, we speculate that the emergence of MGCs in the ovary is for clearing red blood cells resulting from intraovarian bleeding. This further indicates the vascular instability in the ovary treated with BUL+CTX, consistent with a previous report describing vascular injury in human ovary treated with alkylating agents (42). A recent study has demonstrated that ovarian vascular aging contributes to physiological ovarian decline (43). Additionally, Wang et al. has showed the intraovarian vascular endothelial injury in ovary of POI mouse model caused by molybdenum (39). Together with the findings in the present study, we propose that vascular instability may be a common mechanism for ovarian aging. However, p21 deficiency did not prevent the formation of hemosiderin-laden MGCs in BUL+CTX-treated ovaries, indicating that p21 is not required for BUL+CTX-induced vascular instability. Given that MGCs are a type of macrophage, their presence is also linked to inflammation, a well-recognized marker of ovarian aging (44). Consistent with the persistence of MGCs, p21 deficiency had no effect on ovarian inflammation in BUL+CTX-treated mice. These findings provide further evidence that p21 deficiency does not mitigate BUL+CTX-induced ovarian aging.

Increased fibrosis is another hallmark of ovarian aging (28, 45). Previous studies have reported the accumulation of fibrosis in aged ovaries, particularly within the ovarian stroma (28). Although clinical studies have shown that chemical drugs can induce ovarian fibrosis in humans (42), our findings, consistent with our previous report (5), show that BUL+CTX treatment does not increase stromal fibrosis in mice. This difference may be due to the single BUL+CTX injection used in our study, as opposed to the repeated administration of chemotherapy drugs in cancer patients. However, we observed fibrosis in the ovarian cortical region following BUL+CTX treatment, a feature also seen in aged mouse ovaries. Given that the cortical region is enriched with early-stage follicles, it is possible that cortical fibrosis contributes to BUL+CTX-induced ovarian aging. Notably, p21 deficiency did not prevent BUL+CTX-induced cortical fibrosis, providing further evidence that p21 is not required for ovarian aging induced by alkylating agents.

Due to the shrinkage of ovarian follicles, the ovaries of BUL+CTX-treated mice were predominantly occupied by Cyp11a1-positive stromal cells, resembling the phenotype of naturally aged ovaries (6). However, the ratio of Cyp11a1-positive stromal cells to total stromal cells remained stable, regardless of treatment or genotype. Additionally, p21 deficiency did not alter the expression of steroidogenic genes in the ovaries of BUL+CTX-treated mice. Therefore, p21 is not required for the regulation of steroidogenic gene expression, regardless of alkylating agent treatment.

Recent studies have suggested that the accumulation of senescent cells may contribute to ovarian aging (14). It is well established that p21 is a classical marker of cellular senescence (46). To our surprise, p21 deficiency did not appear to alleviate cellular senescence in ovaries treated with BUL+CTX, as shown in the present study. A previous study has demonstrated that p53 deficiency can lead to compensatory upregulation of p16 expression (47). Therefore, it is possible that the upregulation of other CDKIs compensates for the loss of p21, thereby maintaining cell cenescence status. However, none of the tested cell senescence-related CDKIs, including p16, p19, p53, and p27, were upregulated in the ovaries or in other organs of p21-deficient female mice treated with alkylating agents. These results indicate that p21 may not be a crucial mediator of cellular senescence in the ovaries of mice treated with alkylating agents.

Indeed, the conclusion of our present study is unexpected based on findings from other groups. Previous studies have indicated that high expression of p21 is responsible for cell cycle arrest in *in vitro*-cultured ovarian granulosa cells (48–50). High expression of p21 in the ovary has been demonstrated in several mouse models of POI, including naturally aged mice and those treated with cyclophosphamide or doxorubicin (19, 20, 51). A recent study suggested that upregulation of p21 may inhibit the transition from primary to secondary follicles by suppressing ovarian granulosa cell proliferation in a mouse model with Bmi1- and Mel18-deficient ovarian granulosa cells (38). More importantly, the upregulation of p21 has been suggested to be associated with natural ovarian aging in humans (21, 52). However, due to differences in species and methods used to model ovarian aging, the role of p21 may vary and requires further investigation.

Our results need to be interpreted with caution. First, it is still possible that other molecules or pathways not tested in the present study may function to compensate for the loss of p21. In addition, the role of other CDKIs in premature ovarian aging is still needs to be explored by using knockout mouse model, such as p53. Second, this study does not exclude the role of cellular senescence in promoting ovarian aging caused by alkylating agents. Although conclusions from different studies including ours show inconsistent results regarding targeting cellular senescence to alleviate ovarian aging (6, 17, 51, 53). However, integrating cellular senescence with well-established mechanisms of ovarian damage, such as oxidative stress, DNA damage response, and vascular injury, may help identify more promising therapeutic targets for fertility preservation in cancer patients. Third, this study highlights the importance of ensuring proper folliculogenesis in ovaries of female mice treated with alkylating agents. As we have shown, impaired folliculogenesis and overactivation of primordial follicles form a vicious cycle that accelerates ovarian reserve depletion in chemically treated ovaries. Therefore, investigating the disrupted intrinsic signaling pathways which are crucial for proper folliculogenesis in ovaries treated with alkylating agents may represent a valuable research direction for developing future fertility preservation strategies.

**Figure 7:**
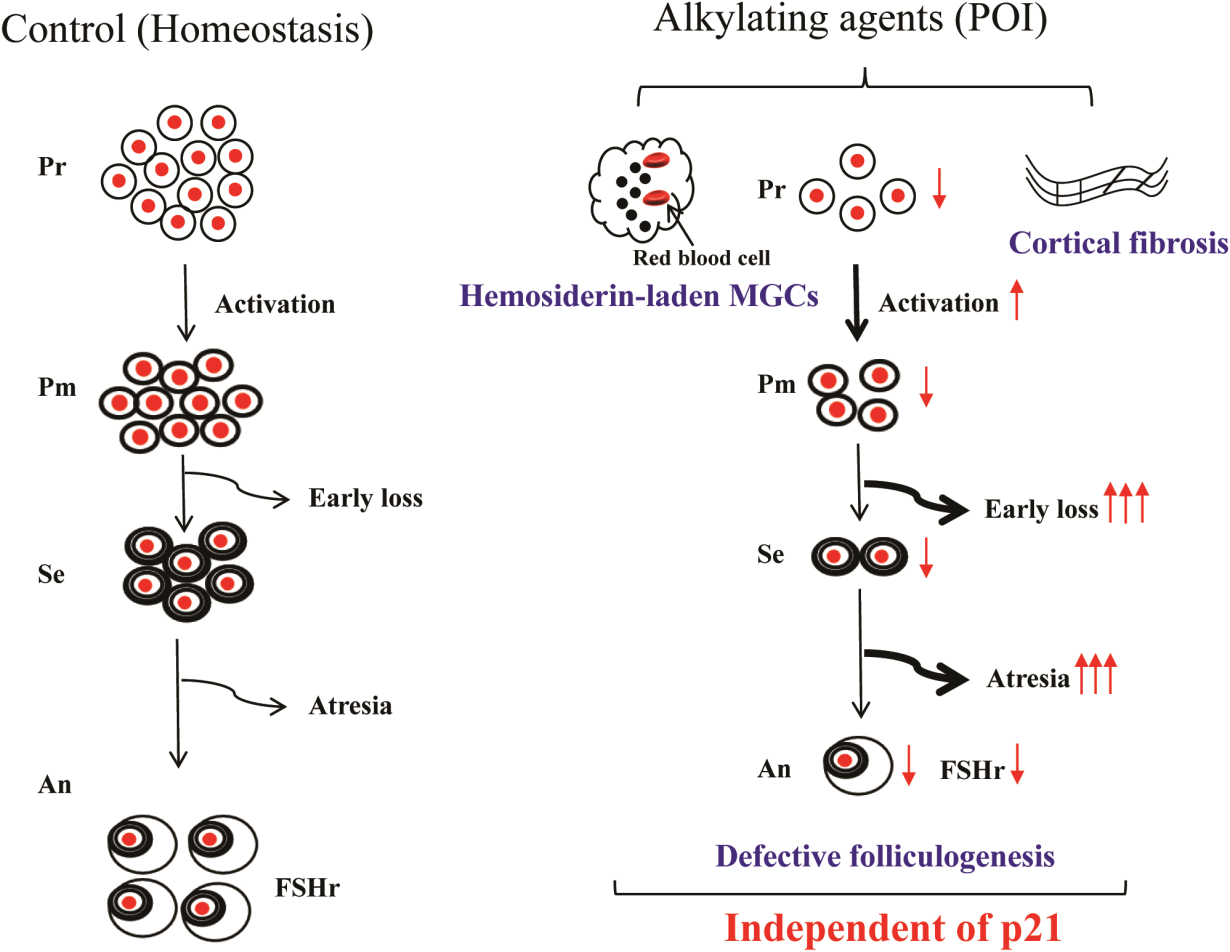
Main findings of the present study. Alkylating agent treatment impaired folliculogenesis, characterized by loss of early-stage follicles, atresia of mid-stage follicles, and poor-quality antral follicles. Consequently, primordial follicle depletion was accelerated by overactivation, accompanied by the presence of hemosiderin-laden multinucleated giant cells and ovarian cortical fibrosis. However, p21 deficiency did not mitigate these alkylating agent-induced changes. Pr, Primordial follicles; Pm, Primary follicles; Se, secondary follicles; An, Antral follicles; POI, premature ovarian insufficiency; MGC, Multinucleated giant cells.

Taken together, we conclude that (1): p21 deficiency did not improve defects in early follicle growth or the abnormal over-activation of primordial follicles induced by BUL+CTX. (2) p21 deficiency did not improve the quality of antral follicles in mice treated with BUL+CTX. (3) p21 deficiency did not reduce the presence of hemosiderin-laden MGCs or fibrosis in the ovarian cortical region (**Figure 7**). This is the first study using p21 knockout mice model to demonstrate that p21 is dispensable for ovarian aging caused by BUL+CTX, suggesting that targeting p21 alone may not mitigate ovarian aging caused by alkylating agents.

## Data Availability

The original contributions presented in the study are included in the article/**Supplementary Material**. Further inquiries can be directed to the corresponding authors.
