# Supplementary material for: Loss of p21 does not protect against premature ovarian insufficiency caused by alkylating agents
Source: Front Endocrinol (Lausanne). 2025 Jul 16;16:1616965. doi: 10.3389/fendo.2025.1616965 (PMC12307192; doi:10.3389/fendo.2025.1616965)
Supplement: Supplementary file 1 [file Image1.pdf]

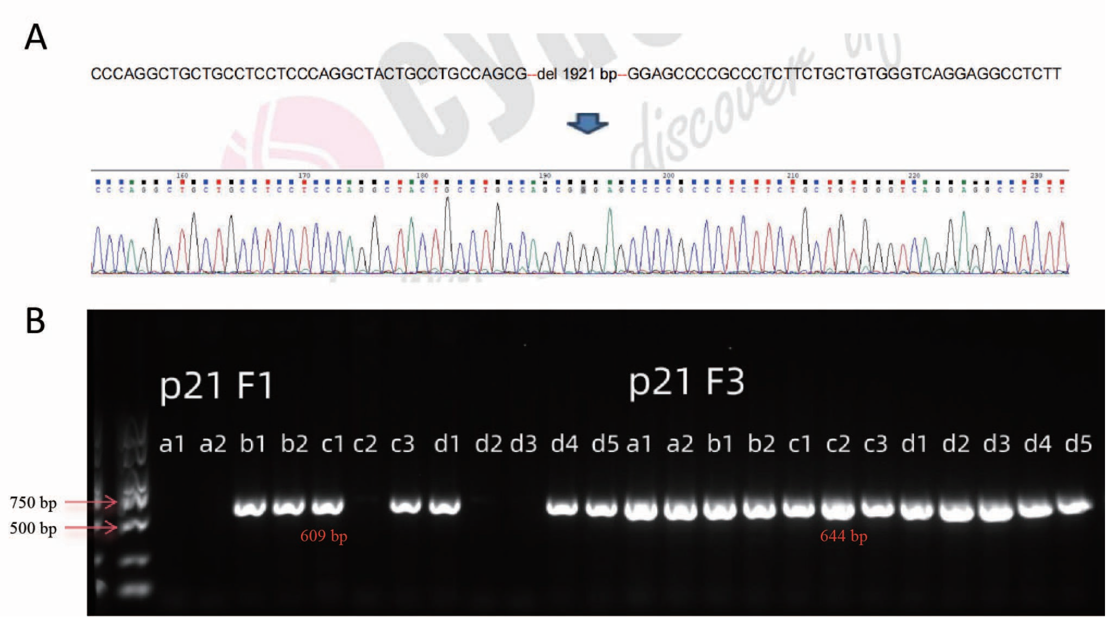

Supplementary figure 1. (A) DNA sequencing confirming the deletion in the p21 gene (Provided by Cyagen Biosciences Company). (B) Genotyping of mice using primer pairs F1/R1 and F3/R1 to detect the knockout (KO) and wild-type (WT) alleles, respectively.

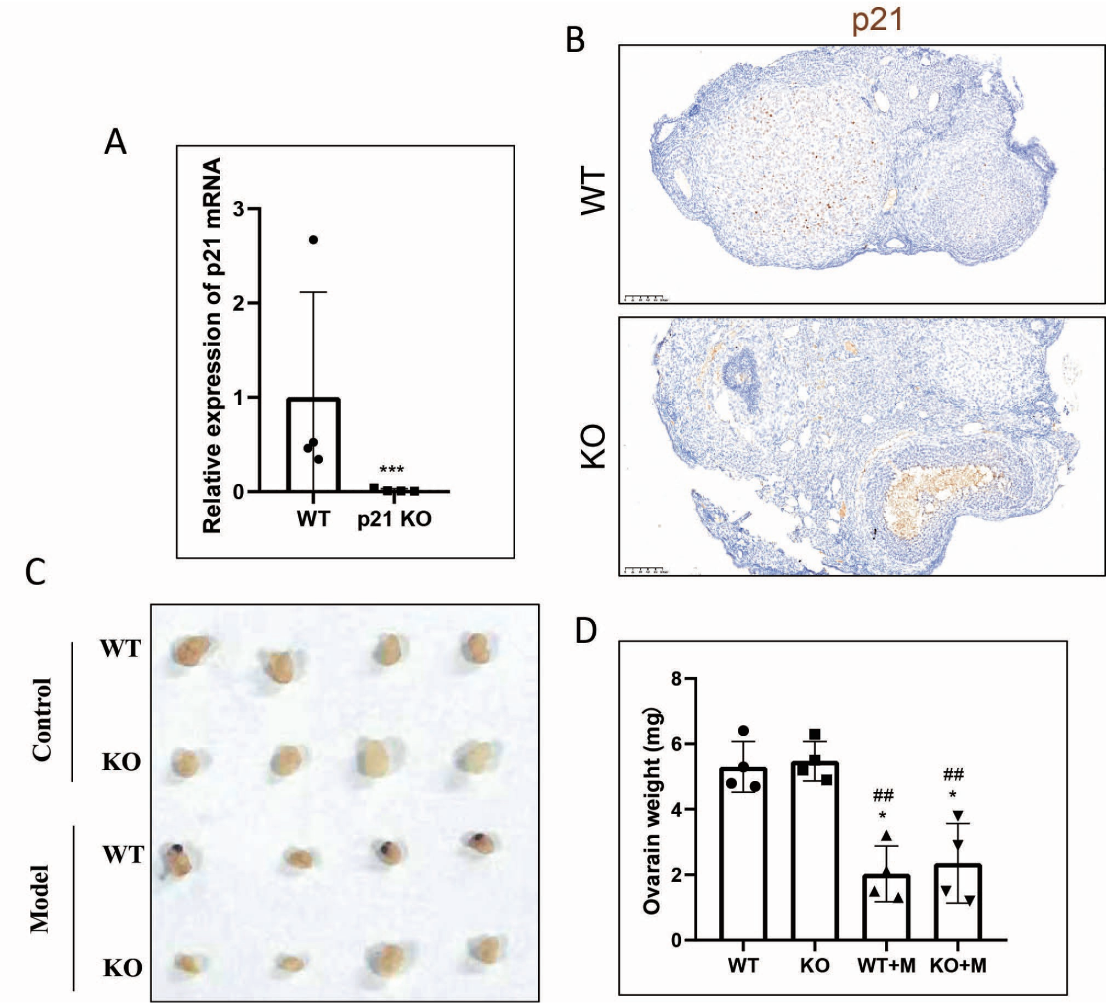

Supplementary Figure 2. p21 deficiency did not affect the reduction in ovarian size caused by BUL+CTX . (A) mRNA expression of *p21* gene in the ovary. N=4 for each group. Statistical analysis was performed using t-student test. (B) Immunohistochemical staining using an anti-p21 antibody. N=3 for each group. (C) Ovarian size. N=4 for each group. Statistical analysis was performed using one-way ANOVA followed by Tukey's multiple comparisons test. (D) Graph depicting ovarian weight. Compared to WT mice, *$p < 0.05$; ***$p < 0.001$. Compared to p21 KO mice, $^{\#}p < 0.05$; $^{\#\#}p < 0.01$. Model: mice treated with BUL+CTX; WT: wild-type; KO: p21 knockout.

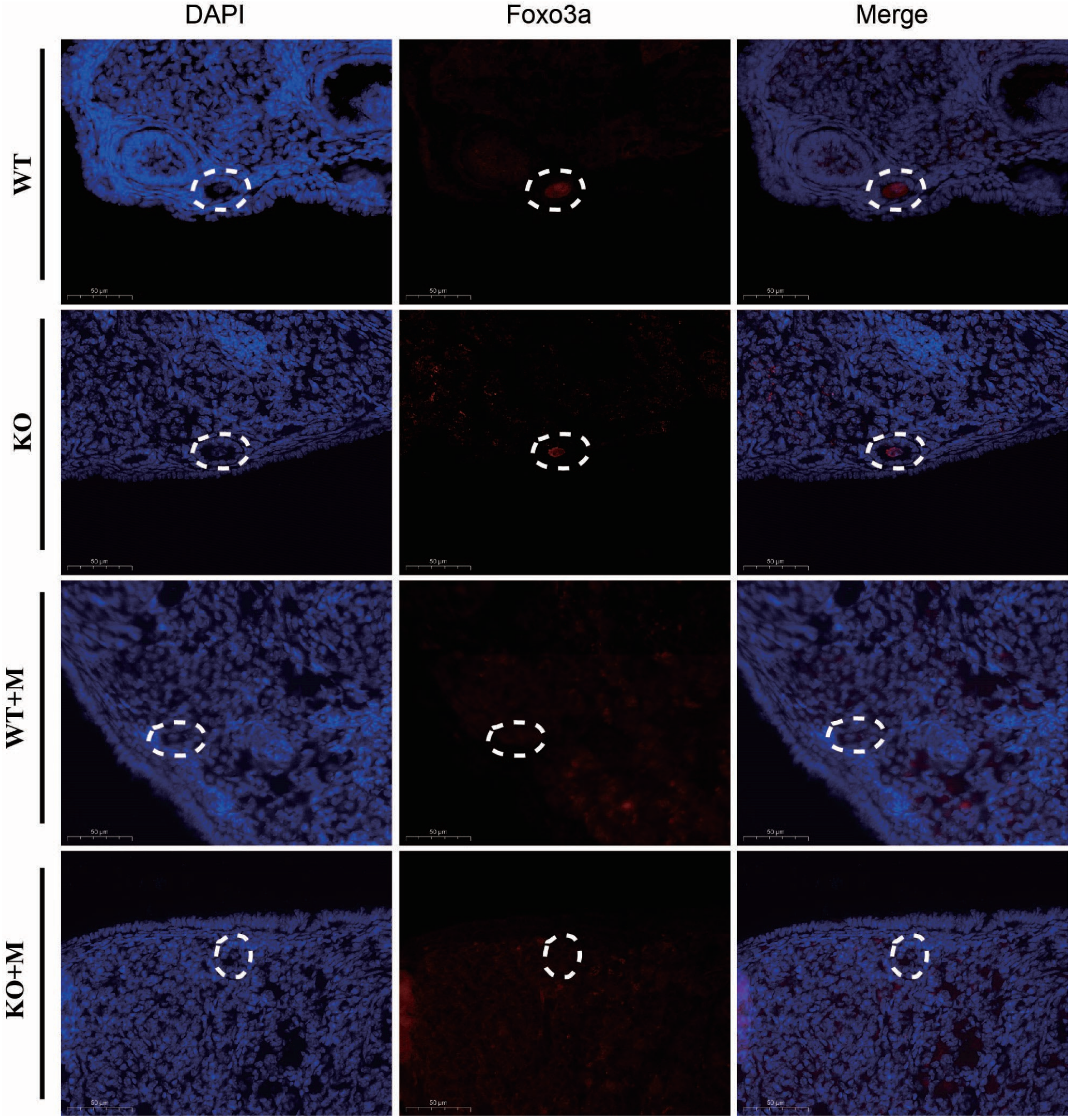

Supplementary Figure 3. p21 deficiency did not prevent over-activation of primordial follicles. Immunohistochemical staining using ant-Foxo3a antibody. N=3 for each group. M: mice treated with BUL+CTX; WT: wild-type; KO: p21 knockout.

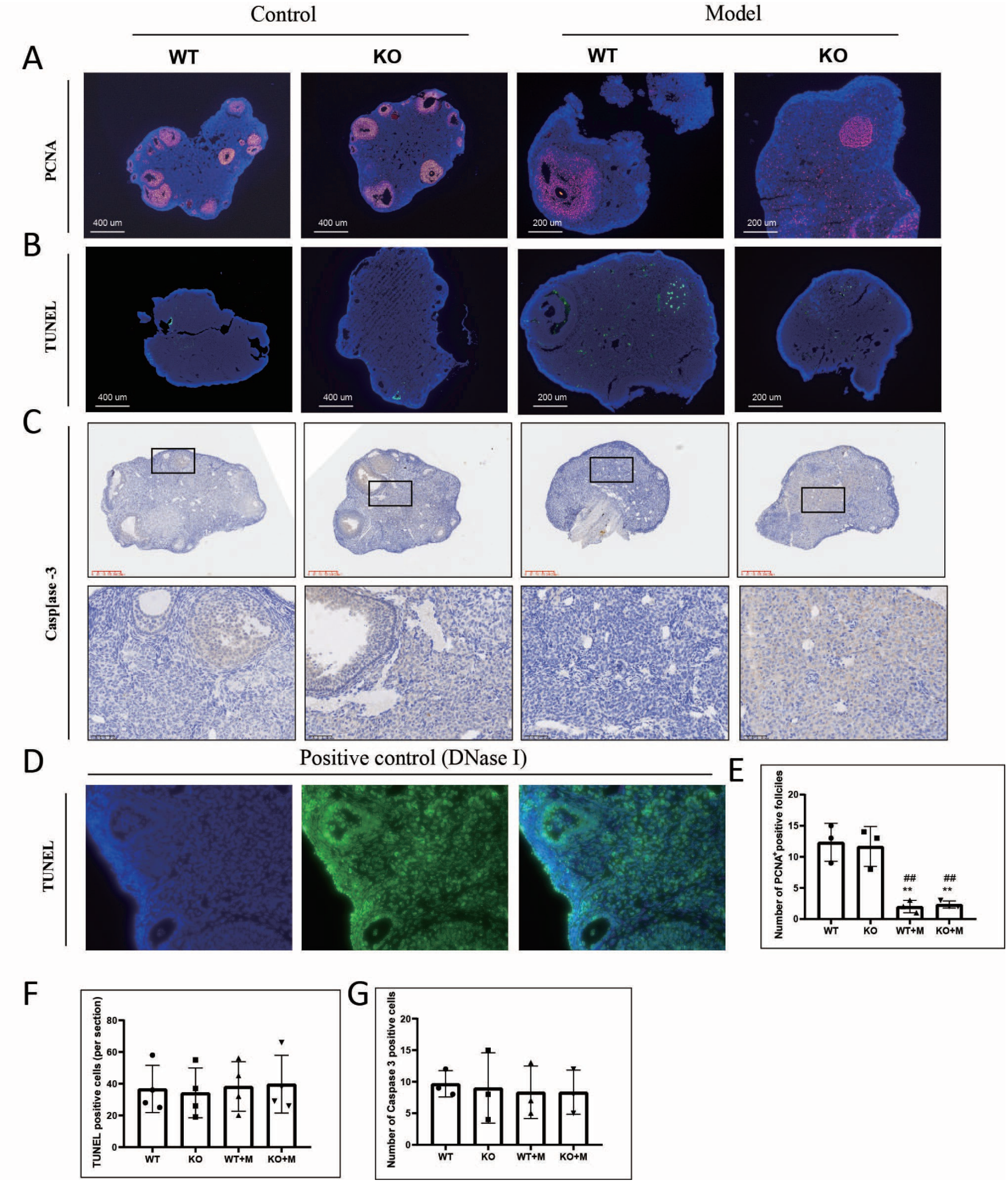

Supplementary Figure 4. p21 deficiency did not improve the reduced number of proliferating ovarian follicles caused by BUL+CTX . (A) Immunofluorescent staining using an anti-PCNA antibody. N=3 for each group. (B) TUNEL assay. N=4 for each group. (C) Immunohistochemical staining using an anti-Caspase 3 antibody. The images below are magnified from the boxed area, N=3 for each group. (D) Positive control of TUNEL assay. The ovarian sections were treated with DNase I. (E) Graph depicting the number of PCNA-positive follicles per section. N=3 for each group. Statistical analysis was performed using one-way ANOVA followed by Tukey's multiple comparisons test. (F) Graph depicting the number of apoptotic cells per section. N=4 for each group. Statistical analysis was performed using one-way ANOVA followed by Tukey's multiple comparisons test. (G) Graph depicting the number of Caspase 3-positive cells per section. N=3 for each group. Statistical analysis was performed using one-way ANOVA followed by Tukey's multiple comparisons test. Compared to untreated WT, $^{**}p < 0.01$;

compared to untreated KO, $^{##}p < 0.01$. Model/M: mice treated with BUL+CTX; WT: wild-type; KO: p21 knockout.

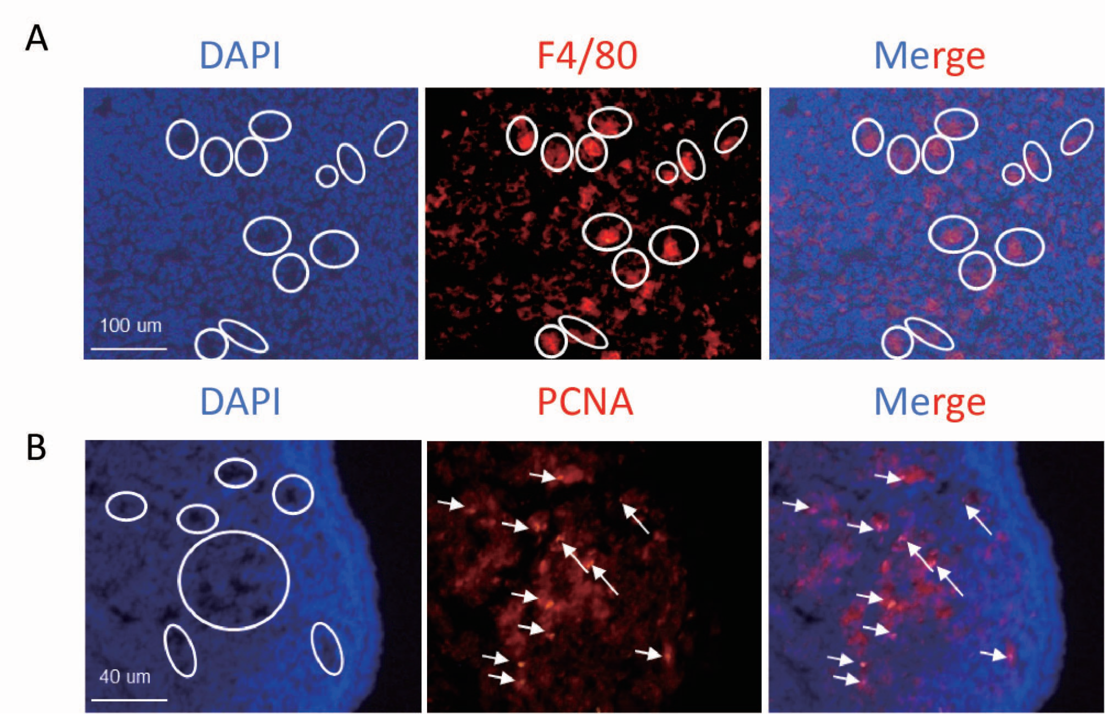

Supplementary Figure 5. Characteristics of MGCs.

(A) Immunofluorescent staining using F4/80 antibody. The white circle highlights MGCs. Due to the large cytoplasm of MGCs, a hole-like appearance is observed following DAPI staining. The "hole" region is occupied by F4/80-positive staining. N=3 for WT or p21 KO mice treated with BUL+CTX. (B) Immunofluorescent staining using a PCNA antibody. The white circle highlights MGCs, and the white arrow indicates PCNA-positive nuclei, which are present in MGCs. N=3 for each group. N=3 for WT or p21 KO mice treated with BUL+CTX.

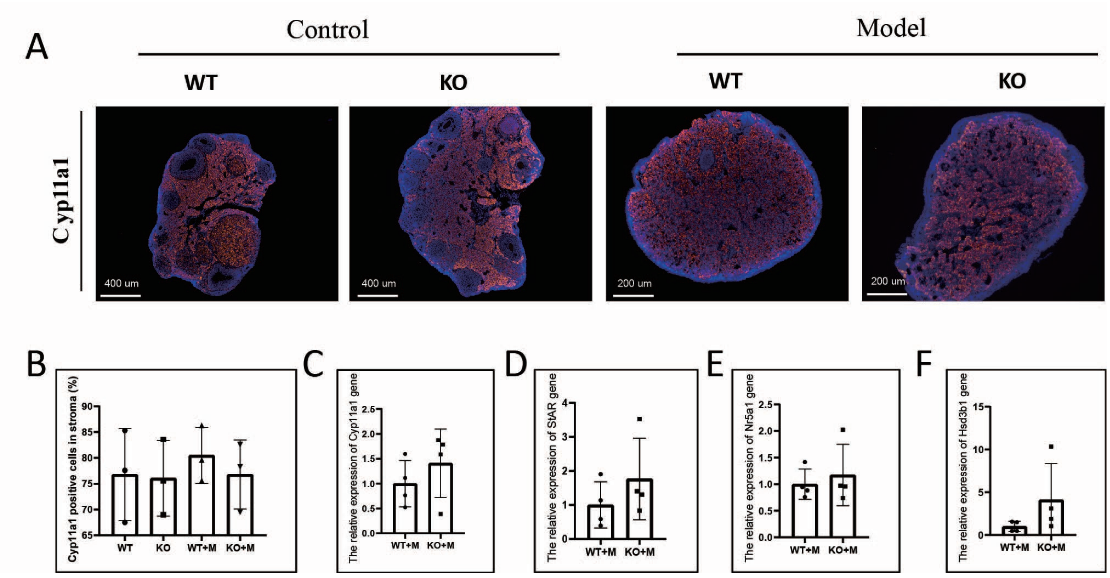

Supplementary Figure 6. p21 deficiency had no impact on characteristics of stromal cells. (A) Immunofluorescent staining using an anti-Cyp11a1 antibody. N=3 for each group. (B) percentage of Cyp11a1-positive cells to stromal cells. N=3 for each group. Statistical analysis was performed using one-way ANOVA followed by Tukey's multiple comparisons test. mRNA expression levels of (C) Cyp11a1, (D) StAR, (E) Nr5a1, and (F) Hsd3b1 in the ovaries. N=4 for each group. Statistical analysis was performed using t-student test. Model/M: mice treated with BUL+CTX; WT: wild-type; KO: p21 knockout.

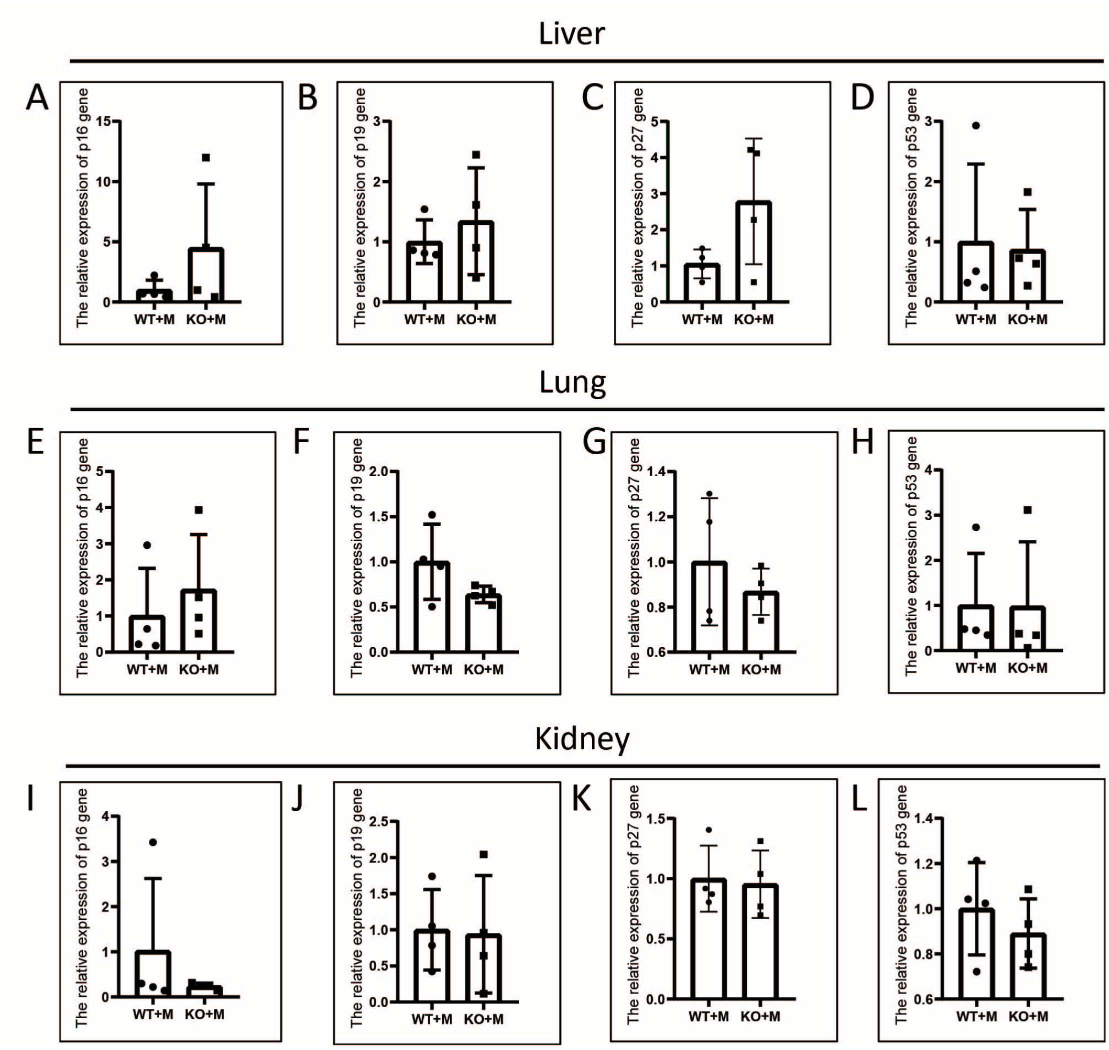

Supplementary Figure 7. p21 deficiency did not affect the expression of other CDKIs in liver, lung, and kidney tissues treated with BUL+CTX. mRNA expression levels of (A) *p16*, (B) *p19*, (C) *p27*, and (D) *p53* in the liver tissues. mRNA expression levels of (E) *p16*, (F) *p19*, (G) *p27*, and (H) *p53* in the lung tissues. mRNA expression levels of (I) *p16*, (J) *p19*, (K) *p27*, and (L) *p53* in the kidney tissues. N=4 for each group. Statistical analysis was performed using t-student test. M: mice treated with BUL+CTX; WT: wild-type; KO: p21 knockout.
