# Supplementary material for: Loss of p21 does not protect against premature ovarian insufficiency caused by alkylating agents
Source: Front Endocrinol (Lausanne). 2025 Jul 16;16:1616965. doi: 10.3389/fendo.2025.1616965 (PMC12307192; doi:10.3389/fendo.2025.1616965)
Supplement: Supplementary file 2 [file Table1.docx]

**Supplementary table 1** The sequence of gRNA-A1, gRNA-A2, primer of DNA sequencing, primer F1, primer R1 and primer F3 are listed.

|  | Sequence |
| --- | --- |
| gRNA-A1 | GAGGCCTTTCCAAAGGACGCTGG |
| gRNA-A2 | GGGCTCCCGTGGGCACTTCAGGG |
| DNA sequencing primer (5’-3’) | TCCAGATTACCTGATCTGCCTGG |
| F1 (5’-3’) | AGCCAGGTAAACCAACACCTTTA |
| R1 (5’-3’) | GGGTTGGGAGGGGCTTAAATAATA |
| F3 (5’-3’) | ATAGTGTGGTGATTGGCAGTAGAG |
