# Supplementary material for: Loss of p21 does not protect against premature ovarian insufficiency caused by alkylating agents
Source: Front Endocrinol (Lausanne). 2025 Jul 16;16:1616965. doi: 10.3389/fendo.2025.1616965 (PMC12307192; doi:10.3389/fendo.2025.1616965)
Supplement: Supplementary file 3 [file Table2.docx]

| **Gene** | **Forward sequence (5’-3’)** | **Reverse sequence (5’-3’)** |
| --- | --- | --- |
| *Gapdh* | ACTTTGGCATTGTGGAAGGG | CATGCCAGTGAGCTTCCCGTT |
| *Cyp11a1* | CTGCCTCCAGACTTCTTTCG | TTCTTGAAGGGCAGCTTGTT |
| *StAR* | TCGCTACGTTCAAGCTGTG | ACGTCGAACTTGACCCATCC |
| *Nr5a1* | TGCAGAATGGCCGACCAG | TGGCGGTAGATGTGGTC |
| *Hsd3b1* | CCTACATTCTGAACTGAGCGGCTGC | GGTCTGTCCTTCCCAGTGATTGATAAAC |
| *FSHr* | TGTGTCATTGCTCTAACAGGGTCT | TTGGGTAGGTTGGAGAACACATC |
| *Cyp19a1* | TCTTGGCTCTACAGAAAGTATGAACG | CGACCTCTGGATACTCTGCGAC |
| *Gdf9* | GTCACCTCTACAATACCGTCCG | TAAACAGCAGGTCCACCATCGG |
| *Bmp6* | CTTTCCTCAACGACGCGGACAT | CCTCAGGAATCTGGGATAGGTTG |
| *Bmp15* | GATTGGAGCGAAAATGGTGAGGC | GCTACCTGGTTTGATGCTAGAGG |
| *AMH* | TGCGCGAGCTGAGTGTAGATCT | TAGCGCGGATTACGGTCAGACT |
| *Foxl2* | TCCGGCATCTACCAGTACATCA | TATTCTGCCAGCCCTTCTTGTT |
| *P16* | GTACCCCGATTCAGGTGATG | GGAGAAGGTAGTGGGGTCCT |
| *p19* | GGAGCTGGTGCATCCTGACGC | TGGCACCTTGCTTCAGGAGCTC |
| *p21* | GTCTTGCACTCTGGTGTCTGAG | TGCGCTTGGAGTGATAGAAA |
| *P27* | GATGTCAAACGTGAGAGTGTCTA | CTGACTCGCTTCTTCCATATCC |
| *P53* | GACCGCCGTACAGAAGAAGAAA | CGGAACATCTCGAAGCGTTTAC |
| *Col1a1* | GGAGAGTACTGGATCGACCCTAAC | ACACAGGTCTGACCTGTCTCCAT |
| *Acta2* | CCCAGACATCAGGGAGTAATGG | TCTATCGGATACTTCAGCGTCA |
| *Il1a* | CGCTTGAGTCGGCAAAGAAAT | TGGCAGAACTGTAGTCTTCGT |
| *IL1β* | TCCAGGATGAGGACATGAGCAC | GAACGTCACACACCAGCAGGTTA |
| *IL6* | CCACTTCACAAGTCGGAGGCTTA | CCAGTTTGGTAGCATCCATCATTTC |
| *IL18* | CCTTTGAGGCATCCAGGACA | GGGAACAGCCAGTGTTCAGT |

**Supplementary table 2**  Primer sequence for RT-qPCR in the present study
